# Transient Viral Activation in Human T Cell Leukemia Virus Type 1-Infected Macaques Treated With Pomalidomide

**DOI:** 10.3389/fmed.2022.897264

**Published:** 2022-05-05

**Authors:** Anna Gutowska, Katherine McKinnon, Sarkis Sarkis, Melvin N. Doster, Massimiliano Bissa, Ramona Moles, James D. Stamos, Mohammad Arif Rahman, Robyn Washington-Parks, David Davis, Robert Yarchoan, Genoveffa Franchini, Cynthia A. Pise-Masison

**Affiliations:** ^1^Animal Models and Retroviral Vaccine Section, Center for Cancer Research, National Cancer Institute, Bethesda, MD, United States; ^2^Department of Microbiological Diagnostics and Infectious Immunology, Medical University of Białystok, Białystok, Poland; ^3^Vaccine Branch Flow Cytometry Core, National Cancer Institute, National Institutes of Health, Bethesda, MD, United States; ^4^HIV and AIDS Malignancies Branch, National Cancer Institute, National Institutes of Health, Bethesda, MD, United States

**Keywords:** human T cell leukemia virus, HTLV-1, pomalidomide, CTL and NK cell activation, macaque model, immune activation

## Abstract

Human T cell leukemia virus type 1 (HTLV-1) persists in the host despite a vigorous immune response that includes cytotoxic T cells (CTL) and natural killer (NK) cells, suggesting the virus has developed effective mechanisms to counteract host immune surveillance. We recently showed that *in vitro* treatment of HTLV-1-infected cells with the drug pomalidomide (Pom) increases surface expression of MHC-I, ICAM-1, and B7-2, and significantly increases the susceptibility of HTLV-1-infected cells to NK and CTL killing, which is dependent on viral *orf-I* expression. We reasoned that by restoring cell surface expression of these molecules, Pom treatment has the potential to reduce virus burden by rendering infected cells susceptible to NK and CTL killing. We used the rhesus macaque model to determine if Pom treatment of infected individuals activates the host immune system and allows recognition and clearance of HTLV-1-infected cells. We administered Pom (0.2 mg/kg) orally to four HTLV-1-infected macaques over a 24 day period and collected blood, urine, and bone marrow samples throughout the study. Pom treatment caused immune activation in all four animals and a marked increase in proliferating CD4^+^, CD8^+^, and NK cells as measured by Ki-67^+^ cells. Activation markers HLA-DR, CD11b, and CD69 also increased during treatment. While we detected an increased frequency of cells with a memory CD8^+^ phenotype, we also found an increased frequency of cells with a Treg-like phenotype. Concomitant with immune activation, the frequency of detection of viral DNA and the HTLV-1-specific humoral response increased as well. In 3 of 4 animals, Pom treatment resulted in increased antibodies to HTLV-1 antigens as measured by western blot and p24Gag ELISA. Consistent with Pom inducing immune and HTLV-1 activation, we measured elevated leukotrienes LTB4 and LTE4 in the urine of all animals. Despite an increase in plasma LTB4, no significant changes in plasma cytokine/chemokine levels were detected. In all cases, however, cellular populations, LTB4, and LTE4 decreased to baseline or lower levels 2 weeks after cessation of treatment. These results indicated that Pom treatment induces a transient HTLV-1-specific immune activation in infected individuals, but also suggest Pom may not be effective as a single-agent therapeutic.

## Introduction

Human T cell leukemia virus type 1 (HTLV-1) is a pathogenic retrovirus that affects 10–20 million people worldwide. It is directly associated with adult T cell leukemia-lymphoma (ATLL), one of the most aggressive T cell malignancies, and HTLV-1-associated myelopathy/tropical spastic paraparesis (HAM/TSP), a progressive neurodegenerative disorder (1–6). HTLV-1 is also associated with other clinical disorders including HTLV-1-associated arthropathy, HTLV-1-associated uveitis, infective dermatitis, and polymyositis (7, 8). While considerable advances have been made in understanding the virology, cell biology, immunology, and pathology of HTLV-1 infection and associated diseases, no highly effective treatments have been realized. In addition, no single biological marker or clinical feature has been identified that accurately predicts disease development, although the HTLV-1 proviral load has been suggested as a possible risk factor for disease development (9–14).

Increases in the HTLV-1 proviral load and persistent infection are likely linked with the virus’ ability to evade the host immune response. We and others have shown that the viral *orf-I* (open reading frame 1) gene product contributes to viral transmission and persistence. The *orf-I* gene product encodes the non-structural protein product p12 which is post-translationally cleaved to the p8 protein (15–17). Both p8 and p12 are dispensable for viral replication *in vitro* (18–21), but have been shown to be essential for viral infectivity/persistence *in vivo* (18, 22). The p12 and p8 proteins counteract natural killer (NK) cells (23) and CD8^+^ cytotoxic T cell (CTL) (22) responses *in vitro* and augment T cell proliferation (24, 25) and viral transmission (26–28). We recently showed that treatment of HTLV-1-infected cells with the drug pomalidomide (Pom) increases surface expression of MHC-I, ICAM-1, and B7-2, and significantly increases the susceptibility of HTLV-1-infected cells to NK killing (29). The effect of Pom on HTLV-1-infected cells is dependent on viral expression, and specifically on the expression of *orf-I*, as demonstrated by a lack of MHC-class I or ICAM-1 upregulation by Pom in human CD4^+^ T cells infected with the HTLV-1 *orf-I* knock-out virus (p12KO). These data suggest that Pom affects the pathway used by *orf-I* proteins to downregulate MHC-I and ICAM-1. By restoring cell surface expression of these molecules, Pom treatment has the potential to reduce virus burden by rendering infected cells susceptible to killing by NK and CTL.

In the present study, we examined the effect of Pom treatment of HTLV-1-infected rhesus macaques to determine if Pom could decrease viral infection *in vivo* via activation of CTL and NK cell activity. We report that Pom treatment caused immune activation as measured by increases in the frequency of activated CD4^+^ and CD8^+^ cells and proliferating memory T cells and NK cells, as well as cells with a Treg phenotype. Consistent with a response to HTLV-1 infection, we also measured an increase in the LTB4 and LTE4 inflammatory markers in urine. Pom treatment also resulted in viral activation as measured by increased detection of viral DNA and HTLV-1-specific antibodies.

## Materials and Methods

### Pomalidomide Treatment of Human T Cell Leukemia Virus Type 1-Infected Cells *in vitro*

Human T cell leukemia virus type 1-infected MT-2 cells were grown as previously described (30). HTLV-1 production was determined by p19Gag antigen detection by a commercially available ELISA in the culture supernatants (Zeptometrix, Buffalo, NY, United States) according to the manufacturer’s instructions. Viral DNA load was determined for established CD4^+^ cell cultures as previously described (31). HTLV-1-infected cells were cultured for 3 days in medium alone, 10 μM of Pom (provided by Bristol Myers Squibb, Summit, NJ, United States), or DMSO as control. For cell-surface staining, cells were incubated with Live/Dead Fixable Blue dye (Thermo Fisher Scientific, Eugene, OR, United States), BV650-conjugated anti-CD54 (BD Biosciences, San Jose, CA, United States), and Alexa 700 Fluor^®^ 700-conjugated anti-CD86 (BD Biosciences) for 30 min at room temperature. For intracellular staining, cells were fixed and permeabilized using eBioscience™ Foxp3 / Transcription Factor Staining Buffer Set (Thermo Fisher Scientific) according to the manufacturer’s instructions prior to their 30 min incubation with the following antibodies: PE-CF594-conjugated anti-Ki-67 (BD Biosciences), PE-Cy7-conjugated anti-human Ikaros (BioLegend, San Diego, CA, United States), and Alexa Fluor^®^ 647-conjugated anti-EZH2 (BD Biosciences). The stained cells were analyzed on a BD FACSymphony A5 analyzer using FACSDiva 8 software and obtained data were analyzed using FlowJo Version 10.6.

### Treatment of Human T Cell Leukemia Virus Type 1-Infected Macaques With Pomalidomide

Four HTLV-1 seropositive and PCR positive rhesus macaques (31) were administered 0.2 mg/kg/day pomalidomide (provided by Bristol Myers Squibb) orally *via* gavage on a schedule of 6 days on drug treatment followed by 1 day off for 24 days. Blood, bone marrow and urine samples were collected on days 5, 12, 18, 24, and 47 of the study. All animals included in this study were colony-bred Indian rhesus macaques (Macaca mulatta). This study was carried out in strict accordance with the recommendations described in the Guide for the Care and Use of Laboratory Animals of the National Institute of Health, Office of Laboratory Animal Welfare, and the United States Department of Agriculture. The animals were housed, and experiments conducted at the National Institutes of Health (Protocol VB033). Animals were cared for in accordance with Association for Assessment and Accreditation of Laboratory Animal Care (AAALAC) standards in an AAALAC-accredited facility (Animal Welfare Assurance A4149-01). All animal care and procedures were carried out under protocols approved by the NCI and/or NIAID Animal Care and Use Committees (ACUC; Protocol number: VB033). Animals were closely monitored daily for any signs of illness, and appropriate medical care was provided as needed. All clinical procedures, including biopsy collection, administration of anesthesia and analgesics, and euthanasia, were carried out under the direction of a laboratory animal veterinarian. Steps were taken to ensure the welfare of the animals and minimize discomfort of all animals used in this study. Animals were fed daily with a fresh diet of primate biscuits, fruit, peanuts, and other food items to maintain body weight or normal growth. Animals were monitored for mental health and provided with physical enrichment including sanitized toys, destructible environments (cardboard and other paper products), and audio stimulation. All procedures were carried out under anesthesia (Telazol, Ketamine/Xylazine or Ketamine HCl) by trained personnel under the supervision of veterinary staff and all efforts were made to ameliorate the welfare and to minimize animal suffering in accordance with the Weatherall report recommendations for the use of non-human primates. Early endpoint criteria, as specified by the IACUC approved score parameters, were used to determine when animals should be humanely euthanized. Animal information is found in Supplementary Table 1.

### Human T Cell Leukemia Virus Serology and Viral DNA Detection

Reactivity to specific viral antigens in the plasma of infected animals was detected with the use of a commercial HTLV-1 western immunoblot assay (MP Diagnostics, Singapore). Monkey anti-measles IgG ELISA (Alpha Diagnostics International, Inc., Antonio, TX, United States) was used to detect antibodies in plasma samples from macaques as described by the manufacturer. HTLV-1 p24 antibodies in plasma samples from macaques were detected and quantified against purified HTLV-1 p24 protein using an ELISA assay (Advanced BioScience Laboratories, Inc., Rockville, MD, United States) according to the manufacturer’s instructions. The plate was read at 450 nm (E-max reader, Molecular Devices, San Jose, CA, United States).

Genomic DNA from PBMC and bone marrow was isolated from animals at baseline and on days 5, 12, 18, 24, and 47 (24 days off drug treatment) of the study using the DNeasy Blood and Tissue Kit (Qiagen, Germantown, MD, United States). One hundred nanograms of DNA were used as templates for the first round of PCR amplification using primers pX-F1, 5′-CCTCGCCTTCCAACTGTCT-3′ and pX-R1, 5′-AGGAAGGAGGGTGGAATGTT-3′. Three microliters of the PCR reaction were used as a template for nested PCR using primers p12-F2, 5′-CGCCTTCCAACTGTCTAGTATAGC-3′and p30-R2, 5′-GGGAGTCGAGGGATAAGGAA-3′. The PCR conditions used were 94°C for 7 min, followed by 35 cycles of 94°C for 30 s, 55°C for 30 s, 68°C for 60 s, and a final extension at 68°C for 7 min, and a hold at 4°C. Platinum High Fidelity PCR SuperMix (Invitrogen, Carlsbad, CA, United States) was used according to the manufacturer’s protocol. Correctly sized amplicons were identified by 1% agarose gel electrophoresis. Sanger sequencing was carried out on the amplicons at the Center for Cancer Research Genomics Core at the National Cancer Institute, NIH, to verify HTLV-1 sequence amplification.

The HTLV-1 proviral load (PVL) was measured by real-time PCR using 5 ng/μl of DNA and the TaqMan Universal PCR Master Mix (Applied Biosystems, Foster City, CA, United States) in the Rotor-Gene Q system (Qiagen). Standard curves were generated by amplification of RNase P gene fragments from HTLV-1-negative genomic DNA with the use of Taqman RNase P Detection Reagents FAM (Applied Biosystems) and the HTLV-1 pX region fragment from pAB-D26 cells (22). The sequences of primers and probes for pX gene were as follows: 5′-CGGATACCCAGTCTACGTGTT-3′, 5’′CAGTAGGGCGTGACGATGTA-3′, and 3′-FAM/CTGTGTA CAAGGCGACTGGTGCC-3′ (32). The reaction conditions were 95°C for 60 s and then 40 cycles of 15 s at 95°C, followed by 60 s at 60°C. HTLV-1 provirus DNA levels were calculated by the following formula:


$$\mathrm{copies}\,\mathrm{of}\,\text{HTLV}-1\,\left(\text{pX}\right)÷\frac{\mathrm{copies}\,\mathrm{of}\,\mathrm{RNase}\,P}{2}\times 100\,\mathrm{cells}$$

Samples with unquantifiable provirus by qPCR (PVL < 0.02% PBMC) were amplified by nested PCR to confirm the presence of viral DNA.

### Leukotriene

Leukotriene B4 (LTB4) and Leukotriene E4 (LTE4) are potent mediators of inflammation, and detectable levels arise from the activation of leukotriene pathways. Plasma samples were collected from whole blood and urine collected by cystocentesis. Urine (2 mls) was centrifuged at 15 K rpm for 1 min at 4°C and supernatant was transferred to a new vial and flash frozen for storage at −80°C until assayed. LTB4 levels in urine and plasma were measured using an ELISA kit (R&D Systems, Minneapolis, MN, United States) following the manufacturer’s instructions. LTE4 levels in urine were measured using an ELISA kit (Cayman Chemical, Ann Arbor, MI, United States) following the manufacturer’s instructions.

### Multiplex Assay of Plasma

Cryopreserved supernatants from plasma collected from rhesus macaques at baseline and days 5, 12, 18, 24, and 47 (24 days off drug treatment) of the study were analyzed using MILLIPLEX MAP Non-Human Primate Cytokine Magnetic Bead Panel kit (Millipore Sigma, St. Louis, MO, United States). The following targets were assayed following the manufacturer’s instructions: IFN-γ, IL-10, IL-15, sCD40L, IL-13, IL-1β, IL-6, IL-8, MIP-1α, MIP-1β, TNF-α, IL-12/23, and IL-18. After thawing the plasma on ice, 25 μl of each were briefly loaded into the well and mixed with 25 μl of assay buffer and 25 μl of magnetic beads. The plates were incubated under agitation at 4° C for 18 h. After washing, 25 μl of detection antibody were added to each well, the plate was incubated, then 25 μl of Streptavidin-Phycoerythrin were added and the plate was washed and mixed with 150 μl of Sheath Fluid. The Median Fluorescent Intensity (MFI) was measured with the Bio-Plex^®^ 200 system with HTF (Bio-Rad Laboratories, Hercules, CA, United States).

### Flow Cytometry Analysis of Rhesus Macaque Samples

Two staining panels were developed for analysis of macaque PBMC and bone marrow from these experiments. A 24-color panel was designed to examine T cell and NK cell phenotypes in response to Pom treatment (surface staining for CD3, CD4, CD8, CD14, CD16, CD20, CD56, NKp44, NKG2a, NKG2d, KIR2DS4, CD45, CD69, CCR2, CD11b, CD11a, CD95, CCR4, IL-15Rα, CD54, CD86, HLA-ABC, HLA-DR, and intracellular Ki-67). A 17-color panel was designed to measure cytotoxic activity in those cell populations (surface staining: CD3, CD4, CD8, CD11b, CD16, CD20, CD45, CD56, NKG2a, intracellular staining for CD107a, perforin, TNF-α, IL-2, granzyme B, and IFN-γ). All antibodies were selected based on cross-reactivity with rhesus macaques and fluorochrome availability. Antibody information for both panels, including clones and fluorochromes, is listed in Supplementary Table 2.

Briefly, frozen cells were thawed, counted, and resuspended (1 × 10^6^ cells/ml) in D-PBS (Thermo Fisher Scientific) and stained with Live/Dead Fixable Blue dye (Thermo Fisher Scientific) and surface stained at room temperature for 30 min. Cells were then fixed with IC Fixation Buffer (Invitrogen), permeabilized with Permeabilization Buffer 10× (Invitrogen) and stained intracellularly for 30 min at room temperature. Samples were washed with D-PBS and resuspended in 1% ultrapure formaldehyde (Tousimis, Rockville, MD, United States). Cytokine production by T cells was measured in cells stimulated with PMA + ionomycin (eBioscience Cell Stimulation Cocktail, Invitrogen) or with peptide pools of 15-mers overlapping by 11 amino acids derived from the entire HTLV-1 Tax (1 μg/ml) and HTLV-1 Gag (1 μg/ml) or medium alone in the presence of brefeldin A (GolgiPlug, BD Biosciences) and monensin (Golgi Stop, BD Biosciences). Following incubation for 6.5 h at 37°C in the presence of 5% CO_2_, cells were subjected to surface and intracellular staining as described above. Samples were acquired on a BD FACSymphony A5 analyzer using FACSDiva 8 software. Data were analyzed using FlowJo Version 10.6. The gating strategy outlined in Supplementary Figure 1 was established using a combination of isotype and fluorescence-minus-one (FMO) controls.

### Statistical Analysis

The parametric student’s paired *t*-test was used and *p*-values < 0.05 were considered significant (*p ≤ 0.05, ^**^p ≤ 0.01, ^***^p ≤ 0.001, ^****^p ≤ 0.0001). The assumption of normality was verified by the Shapiro–Wilk test. Correlation analyses were performed using non-parametric the Spearman-rank correlation method with two-tailed *p*-values calculated (*p* ≤ 0.05).

## Results

### Pomalidomide Increases Surface Marker Expression on Human T Cell Leukemia Virus Type 1-Infected Cells

**FIGURE 1:**
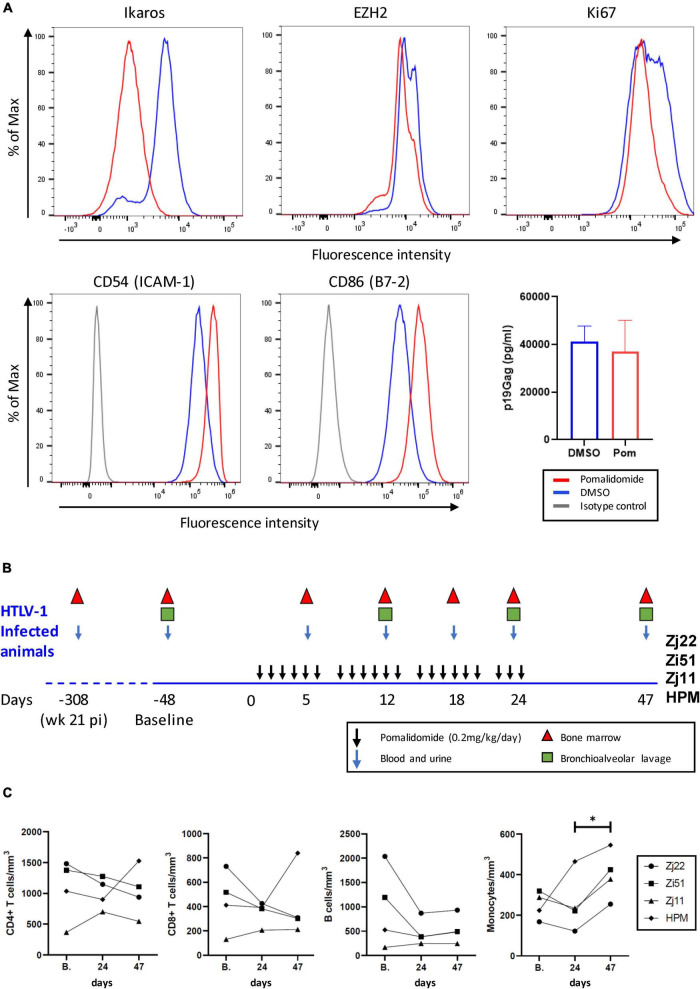
Pomalidomide (Pom) treatment of HTLV-1 transformed cells and HTLV-1-infected rhesus macaques. **(A)** MT-2 cells were cultured for three days in Pom (10 μM) or vehicle (DMSO). Cells were stained for the intracellular factors Ikaros, EZH2, and Ki67, as well as surface-marker expression of CD54 (ICAM-1) or CD86 (B7-2). Representative graphs are shown of percentage of Max vs. Fluorescence Intensity and the supernatant p19Gag concentration from MT-2 cells after three days of culture with Pom (10 μM) or DMSO control. **(B)** Schematic diagram of Pomalidomide treatment and sample collection. Four rhesus macaques previously infected with HTLV-1 (31) were treated orally with 0.2 mg/kg/day of Pomalidomide on a schedule of 6 days of treatment (black arrows) and 1 day off over a 24 day period. Blood and urine (blue arrows), bone marrow (triangles), and bronchioalveolar lavage (squares), were collected as indicated. Twenty-three days after the last drug dose (day 47) final samples were collected. Baseline samples were collected 48 days before starting drug treatment (–48). **(C)** The absolute cell number in cells/mm^2^ for CD3^+^CD4^+^ T cells, CD3^+^CD8^+^ T cells, B-cells, and monocytes were graphed for blood samples for each animal at baseline (B.), day 24 when the last dose of Pom was given, and day 47. Animals are represented as follows: Zj22, circle; Zi51, square; Zj11, triangle; HPM, diamond (*p < 0.05).

The cytotoxic activity of CD8^+^ cells plays a major role in controlling viral infection. We previously demonstrated that the immunomodulatory drug pomalidomide (Pom) increased MHC-1, ICAM-1/CD54, and B7-2/CD86 in HTLV-1 immortalized and transformed T cell lines. Importantly, this upregulation significantly increased their susceptibility to NK cell-mediated cytotoxicity (29). In MT-2 cells, a transformed HTLV-1-infected cell line, we measured surface expression of ICAM-1 and B7-2 on Pom treated cells (10 μM) compared to untreated (Control) or DMSO treated cells (Figure 1A). As we previously reported, ICAM-1 and B7-2 increased in Pom treated MT-2 cells (29). In addition, Pom treatment also caused a decrease in the transcription factors Ikaros and EZH2 in MT-2 cells (Figure 1A), as recently reported (33). Notably, we did not detect a significant decrease in supernatant p19Gag. Because the reduction of Ikaros and EZH2 impact cancer cell growth and increase recognition of infected cells by the host immune system through increased ICAM-1 and B7-2, Pom may have both a direct and an indirect effect on the survival of HTLV-1-infected cells.

### Pomalidomide Treatment Increases the Frequency of Activated CD4^+^ and CD8^+^ T Cells

Four HTLV-1-infected macaques described in a previous study (31) were treated with Pom. All 4 animals had detectable viral DNA and antibody responses by 8 weeks after exposure to wild type HTLV-1, which were sustained over 21 weeks. The animals were infected for nearly a year at the initiation of the current study. To mitigate adverse drug reactions, Pom (0.2 mg/kg/day) was administered orally on a schedule of 6 consecutive days of drug and 1 day off for a total of 24 days. All animals were monitored for an additional 23 days (total of 47 days). The study scheme and sample collection are shown in Figure 1B. Blood samples were taken at baseline, day 24 (corresponding to the last dose of Pom), and day 47 to determine absolute T cell, B cell, and monocyte counts (Figure 1C). Pom treatment was well-tolerated in the 4 macaques. Pom treatment did not significantly change the number of CD4^+^ T cells, CD8^+^ T cells, or B-cells in the four animals, but a marginal increase in monocytes from day 24 to day 47 was noted (final panel, Figure 1C).

**FIGURE 2:**
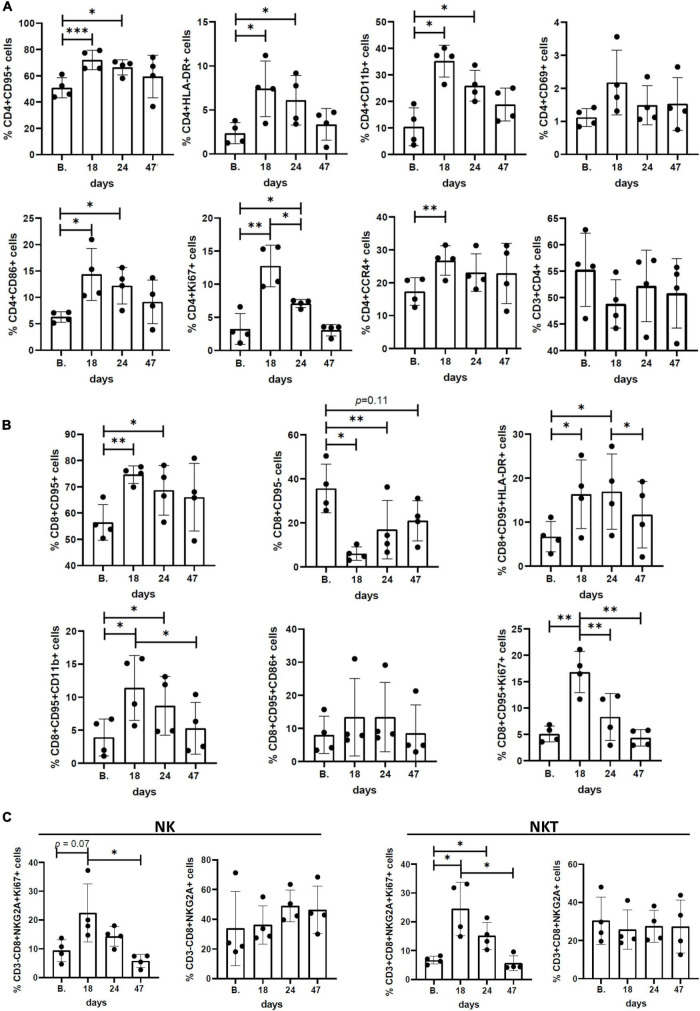
Immunophenotypic profiles and surface/activation markers expression in peripheral blood mononuclear cells (PBMCs) isolated from Pom-treated animals. Graphs represent the frequency (%) of different subsets of **(A)** CD3^+^CD4^+^ cells, **(B)** CD3^+^CD8^+^CD95^+^ cells, and **(C)** CD3^–^CD8^+^NKG2a^+^ (NK) and CD3^+^CD8^+^NKG2a^+^ (NKT) cells at baseline (B.), day 18, day 24 (last dose of Pom), and day 47 (23 days after treatment). The mean ± standard deviation (error bars) is shown and *p*-values (*p < 0.05, ^**^p < 0.01, and ^***^p < 0.001) are given above the columns.

To test if Pom affected specific cell populations in HTLV-1-infected macaques, we used flow cytometry to measure the frequency of specific CD4^+^, CD8^+^, and NK cell populations in PBMC samples from the 4 animals over the course of the study. Supplementary Table 2 provides the antibody clones and fluorochromes used. Frequency of cell populations for all surface markers and intracellular factors are listed for CD4 (Supplementary Table 3), CD8 (Supplementary Table 4), and NK cells (Supplementary Table 5). Flow cytometry analysis showed a clear pattern of cell proliferation and activation in all four animals treated with Pom (Figure 2). In most cases, the highest frequency of CD4^+^ cells with activation markers CD95, HLA-DR, CD11b, and CD69 peaked at day 18 of Pom treatment, and a significant increase in CD4^+^CD95^+^, CD4^+^HLA-DR^+^, and CD4^+^CD11b^+^ was detected at the last dose of Pom (day 24) as compared to baseline before Pom treatment (B.). Consistent with activation, the frequency of CD4^+^ Ki67^+^ cells also peaked at day 18 of Pom treatment and was significantly increased at the last dose of Pom compared to baseline (Figure 2A). We also observed an increase in the frequency of CD4^+^ cells with the co-stimulatory molecule CD86^+^ (B7-2). Interestingly, the frequency of CD4^+^CCR4^+^ cells also increased significantly at day 18 compared to baseline (Figure 2A). CCR4 is a potential marker of T regulatory cells known to suppress T cell responses found on HTLV-1-infected CD4 cells. Likewise, we note a significant increase in the frequency of memory CD8^+^ cells at the last dose of Pom (D24) compared to baseline before treatment (B.). Consistent with increased memory CD8^+^ cells, we observed a significant decrease in the frequency of CD8^+^CD95^–^ cells (Figure 2B). Further, an increased frequency of activated CD8^+^CD95^+^ cells was detected as measured by CD11b^+^ and HLA-DR^+^. Similar to that observed for CD4^+^ cells, a significant increase in proliferating memory CD8 cells (CD8^+^CD95^+^Ki67^+^) was measured at day 18 compared to baseline (Figure 2B). Although the frequency of NK and NKT cells did not significantly change over the course of the study, we did detect an increase in proliferating, Ki67^+^ NK, and NKT cells (Figure 2C). As with CD4^+^ and memory CD8^+^ cells, the highest frequencies of Ki67^+^ NK and NKT cells were measured at day 18. The frequencies of Ki67^+^ NK and NKT cells significantly decreased by day 47.

### Pomalidomide Increases the Frequency of CD4^+^ TNF-α^+^ and CD8^+^ Granzyme B^+^ Cells in Response to Human T Cell Leukemia Virus Type 1 Peptides

**FIGURE 3:**
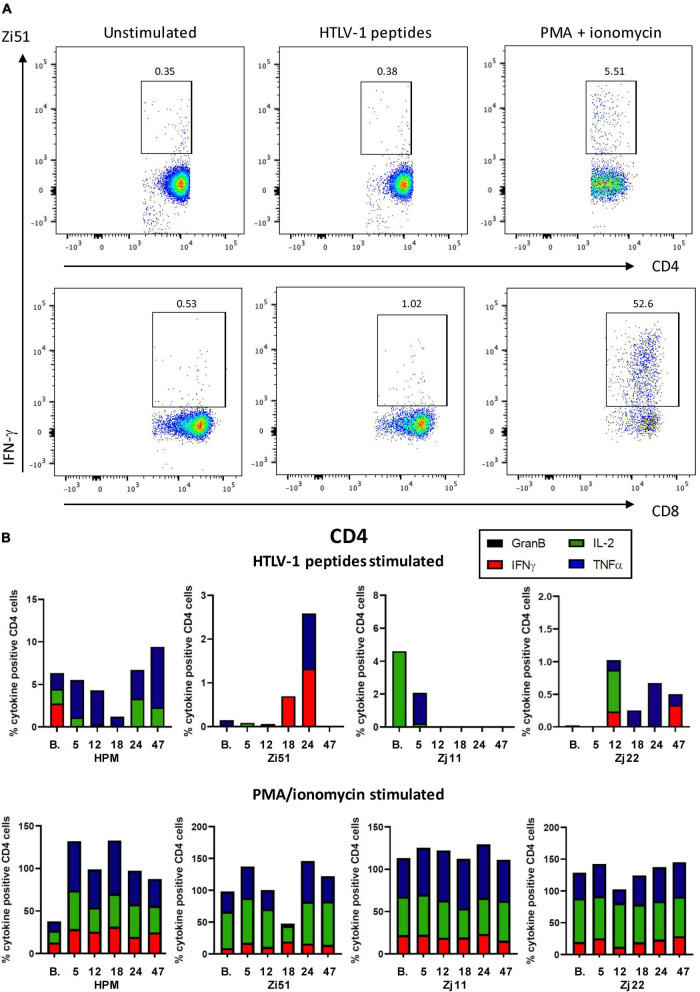
Cell-mediated responses in Pom-treated animals. **(A)** Representative flow cytometric pseudocolor plots showing the frequency of IFN-γ producing CD4^+^ (upper) and CD8^+^ (lower) T cells in bone marrow from unstimulated cultures or after stimulation with HTLV-1 Tax/Gag peptide pools or after stimulated with PMA/ionomycin. **(B)** Plots of the frequency (%) of cytokine producing CD4^+^ cells over the course of the study are shown for each animal. Colors for individual cytokines/granzyme B (GranB) are indicated in the figure. The upper panels represent cultures stimulated with a peptide pool of overlapping 15-mer peptides spanning the HTLV-1 Tax and Gag proteins. The lower panels represent cultures stimulated with PMA/ionomycin.

**FIGURE 4:**
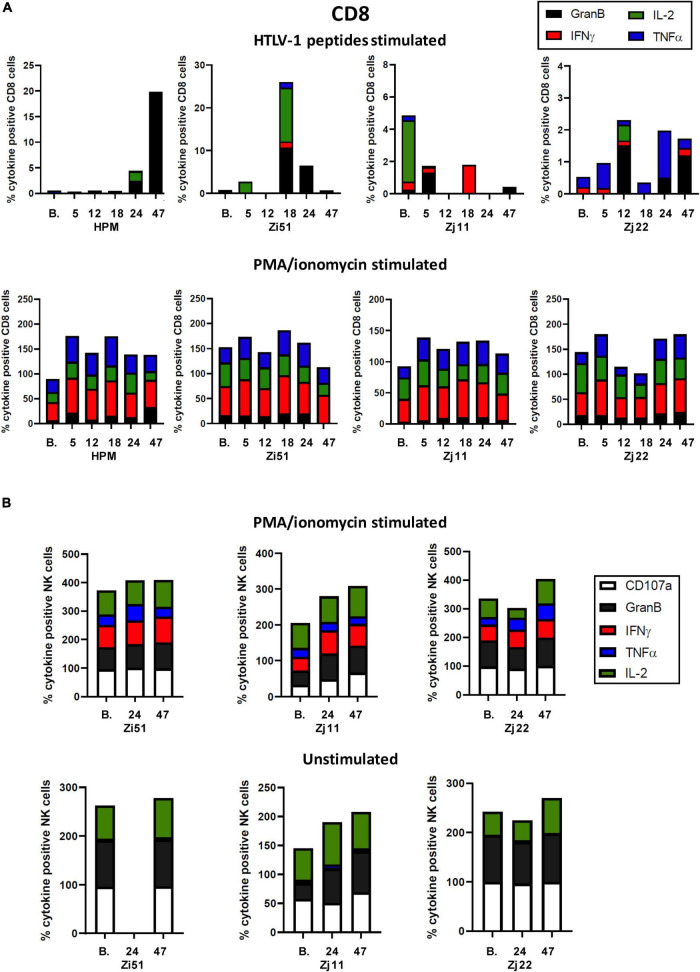
CD8^+^ and NK cell responses in Pom-treated animals. **(A)** Plots of the frequency (%) of cytokine producing CD8^+^ cells over the course of the study are shown for each animal. Colors for individual cytokines/granzyme B (GranB) are indicated in the figure. The upper panels represent cultures stimulated with a peptide pool of overlapping 15-mer peptides spanning the HTLV-1 Tax and Gag proteins. The lower panels represent cultures stimulated with PMA/ionomycin. **(B)** NK cell responses were measured in unstimulated (lower panel) and PMA/ionomycin stimulated (upper panel) cells at baseline (B.) before Pom treatment, day 24 (24) at the last dose of Pom, and day 47 (47).

We next assessed HTLV-1-specific T cell responses by intracellular cytokine staining for IFN-γ, TNF-α, IL-2, and Granzyme B (GranB) in response to a peptide pool of two major antigenic proteins of HTLV-1, Gag and Tax. Representative flow cytometric plots of IFN-γ staining in isolated CD4^+^ and CD8^+^ T cells are shown in Figure 3A. Flow cytometric analysis of the frequency of CD4^+^ and CD8^+^ T cells producing cytokines demonstrated low levels of antigen-specific responses (Figures 3B, 4A). We note that CD4^+^ and CD8^+^ responses to HTLV-1-specific peptides varied in each animal. However, in all four animals, Pom treatment enhanced the percentage of TNF-α^+^CD4^+^ cells in response to HTLV-1-specific peptides at one or more timepoints throughout the study. Similarly, we noted an increase in the percentage of GranB^+^CD8^+^ cells in response to HTLV-1-specific peptides at one or more timepoints (Figures 3B, 4A). As a control, we measured CD4^+^ and CD8^+^ non-specific responses induced by PMA/ionomycin and found a high percentage of responding CD8^+^ cells for IFN-γ, TNF-α, IL-2, and GranB in all animals throughout the study.

To determine if Pom increased NK cell activity, we measured intracellular CD107a, GranB, IFN-γ, TNF-α, and IL-2 in unstimulated and PMA/ionomycin stimulated samples throughout the course of the study. The NK cell count in baseline samples from HPM were too low to accurately measure changes, and this animal was thus excluded from analysis. In unstimulated and PMA/ionomycin stimulated NK cells, animals Zj22 and Zi51 had initial levels of CD107a^+^ and GranB^+^ at >95%, while Zj11 had much lower levels at baseline which increased approximately twofold by day 47 (Figure 4B). We noted CD107a, GranB, and IL-2 positive populations of NK cells in unstimulated cultures, irrespective of Pom treatment. In response to PMA/ionomycin, NK cells responded in a similar manner, again irrespective of Pom treatment, in all three animals. NK cells in PMA/ionomycin stimulated cultures had detectable CD107a, GranB, IFN-γ, TNF-α, and IL-2 positive populations (Figure 4B). This suggests that Pom did not affect the response of NK cells to strong stimuli in HTLV-1-infected macaques.

### Pomalidomide Treatment Is Associated With Increased Human T Cell Leukemia Virus Type 1-Specific Humoral Response and Detection of Viral DNA

**FIGURE 5:**
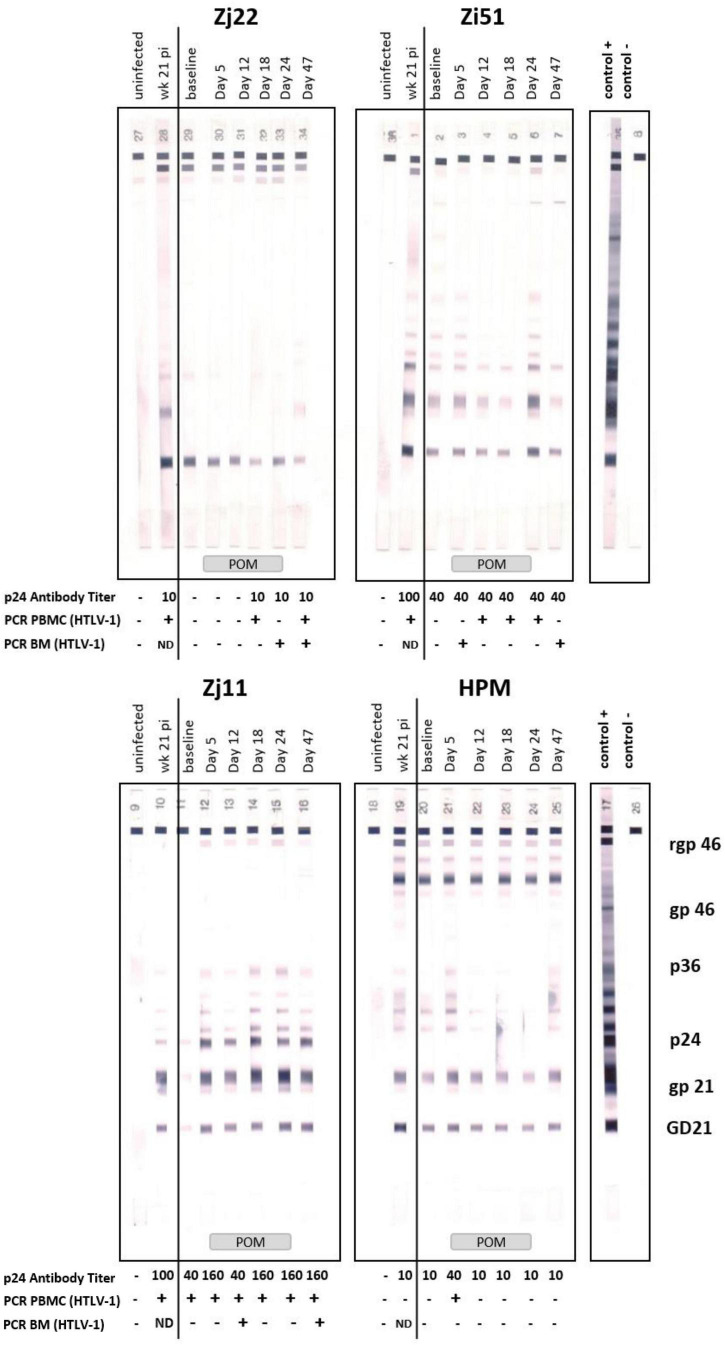
Pomalidomide (Pom) treatment in HTLV-1 non-human primate model. Plasma samples of Zj22, Zi51, Zj11, and HPM prior to infection (uninfected), week 21 post-infection (week 21 pi), at baseline (457 days after infection), and days 5, 12, 18, 24, and 47 were evaluated for reactivity to HTLV-1 antigens using HTLV Blot 2.4 Western Blot Assay (MP Diagnostics, Singapore). Animal IDs are given above each timespoints. Positive and negative control strips are show and HTLV-1 antigens marked. Administration of Pom is indicated at the bottom of the western blot strips. HTLV-1 p24Gag antibody titers were quantified in these same plasma samples using an ELISA assay (Advanced BioScience Laboratories, Inc.). Antibody titers are listed below each timespoints. The minus symbol indicates undetectable levels of p24Gag antibody. Nested PCR for detection of the HTLV-1 pX region was performed on genomic DNA isolated from PBMC or bone marrow (BM) samples at the given timespoints. The plus symbol indicates detection of HTLV-1 pX sequence, the minus symbol indicates undetectable levels of pX and ND means “not done,” as no sample was available.

Next, we assessed changes in HTLV-1-specific antibody responses. Western blot analysis of plasma from infected animals over the course of the study indicate that there was low to no reactivity at baseline. Increased antibody responses were observed in all four animals after treatment with Pom, although the timing of these increases varied (Figure 5). In addition, p24 antibody titers, as measured by ELISA, reflected an increase in 3 of the 4 treated animals and a sustained p24 antibody level in animal Zi51 (Figure 5). To determine if the increase in the antibody response induced by Pom was specific for HTLV-1 or a general increase in reactivity, we measured the IgG antibody titers for measles, as three of the four macaques (Zi51, Zj11, and Zj22) had previously been vaccinated for measles. In contrast to Pom treatment, we detected a decrease in IgG titers for measles over the course of the study (Supplementary Figure 2), thereby demonstrating Pom to have specifically induced increased antibody responses to HTLV-1.

To determine if Pom treatment affected the viral DNA level, we isolated genomic DNA from PBMCs from each animal over the course of the study and used PCR analysis for detection of the pX region of HTLV-1. In this model, the HTLV-1 provirus DNA level in infected animals is <0.02% and often undetectable, and proved unquantifiable in this study. We therefore used nested PCR to detect viral DNA in PBMCs before, during, and after Pom treatment (Figure 5 and Table 1). Macaque Zj11 had detectable levels of viral DNA in PBMCs at baseline and sustained positivity throughout the study. Although negative at baseline, the remaining three animals had detectable HTLV-1 DNA at least once during Pom treatment. Zj11 and Zj22 had detectable viral DNA in PBMCs at day 47, 23 days after suspending Pom treatment. In genomic DNA isolated from bone marrow mononuclear cells, Zj11, Zj22, and Zi51 all had detectable viral DNA at day 47 (Figure 5 and Table 1). No viral DNA could be detected in bronchiolar lavage samples from any of the four animals. Thus, Pom increased the frequency of detection of viral DNA. Taken together, the viral DNA and HTLV-1-specific antibody responses indicate a reactivation of viral expression and/or proliferation of infected cells upon treatment with Pom.

**TABLE 1:** Human T cell leukemia virus type 1 (HTLV-1) DNA detection in infected macaques after Pomalidomide treatment.

pX region							
Material*	Animal	Baseline	Day 5	Day 12	Day 18	Day 24	Day 47
PBMC	Zj22				+		+
	Zi51			+	+	+	
	Zj11	+	+	+	+	+	+
	HPM		+				
BM	Zj22					+	+
	Zi51		+				+
	Zj11			+			+
	HPM						

*PBMC-peripheral blood mononuclear cells; BM-bone marrow cells. (+) indicates the sample was positive by nested PCR for the 755 base pair pX region of HTLV-1.

### Inflammatory Response

**FIGURE 6:**
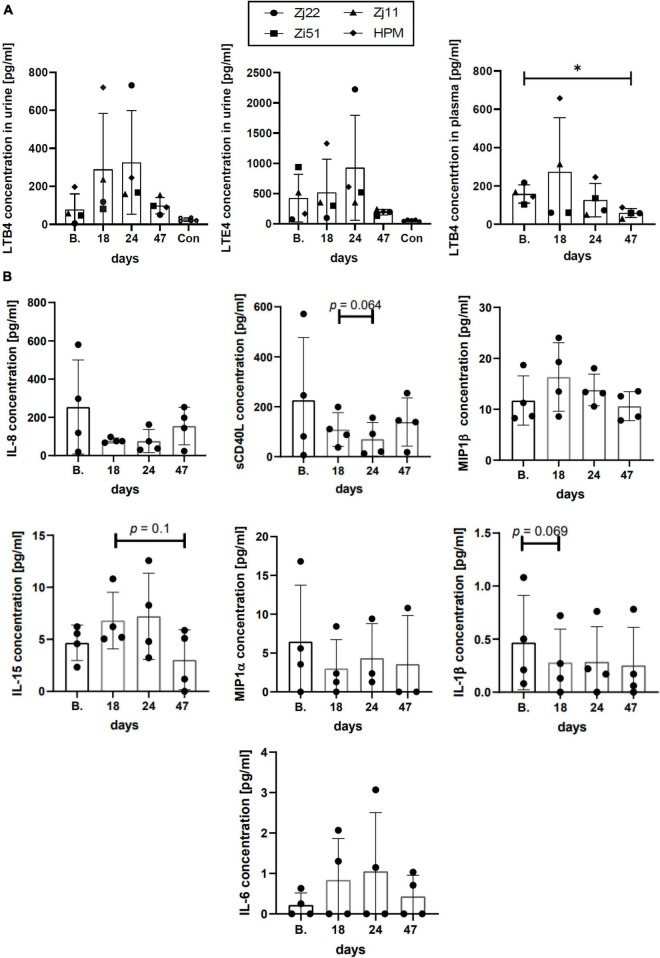
Inflammatory response. **(A)** Leukotriene concentrations in urine and plasma. The lipid mediators LTB4 and LTE4 play a pivotal role in acute and chronic inflammation. The concentration of LTB4 (in urine and plasma) and LTE4 (in urine) were measured over the time of infection using commercial ELISA kits. Concentrations of LTB4 and LTE4 in the urine of uninfected control animals are included (Con). Symbols represent individual animals at given timespoints: Zj22, circle; Zi51, square; Zj11, triangle; HPM, diamond. The mean ± standard deviation (error bars) is shown (*p ≤ 0.05). **(B)** Cytokine/Chemokine levels in plasma. The levels of cytokines/chemokines in the plasma of HTLV-1-infected animals at the given timespoints throughout the study were measured with MILLIPLEX MAP Non-Human Primate Cytokine Magnetic Bead Panel kit (Millipore Sigma). Graphs represent the results at baseline (B.), day 18, day 24 when the last dose of Pom was administered, and day 47 for cytokines/chemokines detected in all four animals. The mean ± standard deviation (error bars) is shown and *p*-values < 0.15 are given above the columns.

Leukotrienes are lipid mediators associated with several inflammatory diseases which have been shown to be biomarkers for HTLV-1 infection. Leukotrienes LTB4 and LTE4, the latter a stable metabolite of cysteinyl leukotrienes, were previously shown to be increased in HTLV-1-infected individuals compared to controls and correlated with viral DNA load (34). Since leukotrienes are detectable in plasma and/or urine, samples were collected from both sources before, during, and after Pom treatment to measure LTB4 and LTE4 levels. The levels of LTB4 concentration in the urine of uninfected animals were in the range of 17–36 pg/ml (Figure 6A and Supplementary Table 6). Three of the four HTLV-1-infected macaques had high urine LTB4 levels ranging from 47 to 197 pg/ml. One macaque, Zj22, had low LTB4 levels at baseline (4.9 pg/ml) but upon Pom treatment the level of LTB4 dramatically increased to 731.7 pg/ml at day 24 of treatment (Figure 6 and Supplementary Table 6). Likewise, LTB4 levels increased dramatically in HPM at day 18 of Pom treatment to 720.7 pg/ml, and to a lesser extent in Zj11 to 236.1 pg/ml at day 18 and in Zi51 to 166.9 pg/ml at day 24. LTB4 increases followed a similar profile in the plasma of Pom treated animals (Figure 6A and Supplementary Table 6).

Leukotriene E4, which is only detectable in urine, showed fluctuation upon treatment with Pom. As with LTB4, animals Zi51, Zj11, and HPM had higher baseline levels of LTE4 in urine than uninfected animals, at a range of 160–958 pg/ml compared to a range of 45–119 pg/ml, respectively (Figure 6A and Supplementary Table 6). Zj22 had baseline urine LTE4 levels of 70 pg/ml, which is consistent with the range in uninfected macaques. Similar to what we observed for LTB4, LTE4 urine levels increased during Pom treatment. For all Pom treated animals, the urine levels of LTE4, as well as LTB4, dropped 23 days (day 47) after stopping drug treatment. Indeed, a significant difference was detected between the plasma LTB4 levels at baseline compared to day 47 (Figure 6A). Therefore, consistent with above results, the leukotriene levels suggest that Pom causes a reactivation of HTLV-1 in infected macaques resulting in increased inflammation, which resolves when Pom treatment is stopped.

Next, we measured cytokine and chemokine levels in plasma at baseline, during Pom treatment, and 23 days after stopping Pom treatment (day 47). Of the twelve cytokine/chemokines assessed, we detected IL-1β, MIP-1α, MIP-1β, IL-6, IL-8, IL-15, and sCD40L. Unlike what we observed for leukotriene levels, Pom did not cause consistent changes in cytokine/chemokine levels in HTLV-1-infected macaques (Figure 6B and Supplementary Table 7). Interestingly, there was a positive correlation in three of the four macaques (Zj22, Zj11, HPM; *p* = 0.033, *p* = 0.003, *p* = 0.033, respectively) between IL-15 levels and Ki-67^+^ memory CD8^+^ cells. IL-15 is known to stimulate antigen-specific CD8 cells and NK cells and used in immunotherapy (35). Together, these results provide evidence that Pom causes immune activation in HTLV-1-infected macaques.

## Discussion

Viruses have evolved a variety of mechanisms to evade the host immune response to establish a persistent infection. For HTLV-1, immune evasion may be associated with a high viral replication rate that can lead to a high proviral load, which, in turn, is associated with disease manifestation (11, 12, 14, 36, 37). We and others have shown that the HTLV-1 *orf-I* gene plays an important role in cellular proliferation and viral persistence (38).

The Orf-I protein interacts with the heavy chain of major histocompatibility complex class I (MHC-I) and reroutes it to proteasomal degradation (39), causing a decrease in cell surface MHC-I expression and suboptimal recognition of infected cells by cytotoxic T cells (22, 29). Orf-I also down regulates ICAM-1 and ICAM-2, reducing NK cell recognition (23, 29). At the plasma membrane, the p8 protein isoform of Orf-I is recruited to the immunological synapse, and, upon antigen stimulation, downregulates proximal T cell receptor (TCR) signaling, thus causing T cell anergy (27). In addition, the p8 protein is transferred from cell to cell by cellular conduit and increases T cell adhesiveness and HTLV-1 transmission (22, 26, 40–43). Recently, we found that *in vitro* the immunomodulatory drug pomalidomide could counter the Orf-I protein’s down-regulation of MHC-1, B7-2, and ICAM-1 and allow CTL and NK cell killing (29).

The thalidomide analogs lenalidomide and pomalidomide are used in the treatment of multiple myeloma, and pomalidomide was recently approved in the United States for the treatment of Kaposi sarcoma (44). Recently, a phase II clinical trial using lenalidomide to treat 26 patients with relapsed/recurrent ATL (15 acute, 7 lymphoma, 4 chronic) was conducted (45). Lenalidomide (Len) demonstrated tolerable toxicity with significant anti-leukemic activity, leading to approval of lenalidomide in the treatment of relapse/refractory ATL in Japan. However, in a phase II study of lenalidomide in 4 patients with relapsed/refractory ATL performed in the United States, lenalidomide showed no clinical activity (46).

Our *in vitro* results confirm earlier findings that Pom can have both a direct and an indirect effect on HTLV-1-infected cells (29, 33, 47). Treatment of HTLV-1-transformed cells with Pom causes a decrease in transcription factors EZH2, Ikaros, Aiolos, STAT3 and IRF4, which are involved in cell survival and signaling pathways. Pom treatment resulted in reduced HTLV-1-infected cell growth. Although Ikaros and EZH2 decreased with Pom treatment, we did not detect a significant decrease in supernatant p19Gag, a measure of virus production. In addition to a direct effect on cell growth, the ability of Pom to increase surface expression of MHC-1, ICAM-1, and B7-2 on HTLV-1-infected cells increases the possibility of recognition of infected cells by the host immune response *via* CTL and NK cells. We treated HTLV-1-infected macaques with Pom in order to test whether or not the drug could activate the host immune response to HTLV-1 and lower the viral burden.

Because the DNA viral load is below the level of detection in these animals, we could not quantify changes in viral DNA load. Surprisingly, using nested PCR, we found that Pom treatment increased the frequency of detection of viral DNA in PBMCs of treated animals. This suggests that *in vivo* Pom may induce proliferation of infected cells or viral transmission. Further, Pom treatment transiently augments the humoral response to the virus and inflammatory markers. With Pom treatment, we detected increased HTLV-1-specific antibodies and LTB4 in plasma as well as elevated LTB4 and LTE4 in urine. Leukotrienes are lipid mediators involved in several inflammatory disorders and have been shown to be elevated in the plasma of HTLV-1-infected individuals, with LTB4 concentration positively correlating with the HTLV-1 proviral load (34). In addition, LTB4 was found to be secreted from HTLV-1-infected CD4^+^ T cells in a Tax-dependent manner and that LTB4 in culture supernatants favors cell to cell interaction and viral spread (48). Further, approximately 3-5% of HTLV-1 infected individuals will develop the inflammatory disease HTLV-1-associated myelopathy/tropical spastic paraparesis (HAM/TSP). Although mixed results have been reported, HAM/TSP patients, especially those from Japan, seem to have a good response to steroid treatment (49–52). It would be interesting to test if the combination of Pom or Len with steroids could reduce pain and improve or stabilize motor function in these patients. Indeed, lenalidomide plus low-dose dexamethasone until progression is the standard of care for newly diagnosed multiple myeloma patients (53, 54).

Consistent with viral activation or proliferation of infected cells, we measured increased immune activation and noted CD4, CD8, and NK cell proliferation, which peaked at 18 days following Pom treatment. We also measured increases in activated CD4^+^ and CD8^+^ cells. Interestingly, the CD8^+^ cells show a memory phenotype expressing CD95^+^, CD11b^+^, and HLA-DR^+^. CD4^+^CCR4^+^, which are markers associated with T regulatory (Treg) cells, also increased. A declining response seen at day 24 may possibly be explained by the last dose of Pom increasing Treg cells, although further studies are needed to confirm this hypothesis. When we measured changes in HTLV-1-specific responses with Pom treatment, we detected increases in the percentage of CD4^+^ producing the inflammatory cytokine TNF-α^+^ and cytotoxic CD8^+^ expressing Granzyme B in all four animals. However, the responses varied from animal to animal, and it appeared that the Pom regimen used in our study was not sufficient to maintain immune activation against the virus as most responses decreased by the last dose of Pom and remained lower after suspending treatment. The decreased activation is possibly linked to an increase in the frequency of Treg cells. More studies are needed to further characterize the T cell response and test whether a different dosing regimen would be more effective.

Together, our results suggest that pomalidomide can enhance the immune response to HTLV-1 infection, but this response is not maintained. Since Pom and Len are well-tolerated, combinatorial treatment with other chemotherapeutics or monoclonal antibodies could be more effective. In the case of multiple myeloma, the combination of Len with proteasome inhibitors and other drug classes such as HDAC inhibitors has been successful (55). Indeed, in a case report by Oka and colleagues, Len demonstrated potential for maintenance therapy of ATL. When daily low-dose Len was given following LSG5 chemotherapy with mogamulizumab, an acute ATL patient maintained complete remission with no recurrence for at least 24 months. Further, Len treatment resulted in long-term increases in the number of cytotoxic T cells, CD4^+^ cells, and NK cells (56). This cell proliferation is consistent with what we found here for Pom in the macaque model. Moreover, we recently found that CTL and NK cells are critical for the establishment of persistent HTLV-1 infection (31). Thus, Pom or Len treatment able to activate NK cells combined with the use of monoclonal antibody treatment could potentially also enhance ADCC in ATL patients.

## Data Availability Statement

The original contributions presented in the study are included in the article/Supplementary Material, further inquiries can be directed to the corresponding author.

## Ethics Statement

The animal study was reviewed and approved by the NCI and/or NIAID Animal Care and Use Committees (ACUC; Protocol number: VB033).

## Author Contributions

CP-M and GF designed the study, analyzed the data, and wrote the manuscript. AG performed experiments and analyzed the data. KM, MD, SS, MB, RM, RW-P, JS, and MR performed the experiments. AG, KM, MD, SS, MB, RM, RW-P, JS, MR, DD, and RY contributed to the interpretation of the data and critical review of the manuscript. All authors contributed to the article and approved the submitted version.

## Author Disclaimer

The content of this publication does not necessarily reflect the views or policies of the Department of Health and Human Services, nor does mention of trade names, commercial products, or organizations imply endorsement by the U.S. Government.

## Conflict of Interest

RY reports receiving research support from Celgene (now Bristol Myers Squibb) through CRADAs with the NCI. RY also reports receiving drugs for clinical trials from Merck, EMD-Serano, Eli Lilly, and CTI BioPharma through CRADAs with the NCI, and he has received drug supply for laboratory research from Janssen Pharmaceuticals. RY and DD are co-inventors on US Patent 10,001,483 entitled “Methods for the treatment of Kaposi’s sarcoma or KSHV-induced lymphoma using immunomodulatory compounds and uses of biomarkers”. RY was also a coinventor on patents on a peptide vaccine for HIV and on the treatment of Kaposi sarcoma with IL12, and an immediate family member of RY was a co-inventor on patents related to internalization of target receptors, on KSHV viral IL-6, and on the use of calreticulin and calreticulin fragments to inhibit angiogenesis. All rights, title, and interest to these patents have been or should by law be assigned to the U.S. Department of Health and Human Services; the government conveys a portion of the royalties it receives to its employee inventors under the Federal Technology Transfer Act of 1986 (P.L. 99-502). The remaining authors declare that the research was conducted in the absence of any commercial or financial relationships that could be construed as a potential conflict of interest.

## Publisher’s Note

All claims expressed in this article are solely those of the authors and do not necessarily represent those of their affiliated organizations, or those of the publisher, the editors and the reviewers. Any product that may be evaluated in this article, or claim that may be made by its manufacturer, is not guaranteed or endorsed by the publisher.

## Supplementary Material

Supplementary Figure 1 Gating strategy. The gating strategy used for flow cytometry evaluation of the different **(A)** T cell subsets, **(B)** NK cells, **(C)** NKT cells, and **(D)** intracellular cytokine staining.

Supplementary Figure 2 Measles IgG antibody titers. Zi51 (square), Zj11 (triangle) and Zj22 (circle) were immunized for measles prior to the start of any study. HPM (diamond) was not immunized for measles. A graph of the monkey anti-measles IgG as measured by ELISA (Alpha Diagnostics International, Inc) is graphed for each animal at baseline (B.), day 18, day 24, and day 47 (23 days post Pom treatment).

Supplementary Table 1 Animal history. The four animals used in this study were infected with HTLV-1 in a previous study investigating cell populations in viral infection (31). All macaques were infected for more than 350 days before treatment with Pom (0.2 mg/kg/day). HTLV-1 infection was confirmed by Western blot and PCR. One day prior to exposure to HTLV-1, macaques were treated with either clodronate at 5 mg/kg (Clodrosome^®^) or liposome control (Encapsome^®^, Encapsula NanoSciences LLC, Brentwood, Tennessee).

Supplementary Table 2 Antibodies used for non-human primate flow cytometry. Antibodies used for non-human primate flow cytometry, including clones, fluorochromes, and source.

Supplementary Table 3 Changes in phenotypic profiles of CD4+ cells with Pom treatment.

Supplementary Table 4 Changes in phenotypic profiles of CD8+ cells with Pom treatment.

Supplementary Table 5 Changes in phenotypic profiles of NK cells with Pom treatment.

Supplementary Table 6 Leukotriene levels in plasma and urine.

Supplementary Table 7 Plasma cytokine and chemokine levels. Cytokine and chemokine levels measured in pg/ml are listed for each animal prior to infection with HTLV-1 (uninfected), after infection (baseline), during the course of Pom treatment (days 5, 12, 18, 24), and 23 days after Pom administration (day 47). Cytokines/chemokines not in the table: TNF-α, IL-10, IFN- γ, Il-12/23, and IL-13 were below detectable levels in animals Zj22, Zj11, and HPM at all timepoints.
