# Supplementary figures and images for: Transient Viral Activation in Human T Cell Leukemia Virus Type 1-Infected Macaques Treated With Pomalidomide
Source: Front Med (Lausanne). 2022 May 5;9:897264. doi: 10.3389/fmed.2022.897264 (PMC9119179; doi:10.3389/fmed.2022.897264)

A

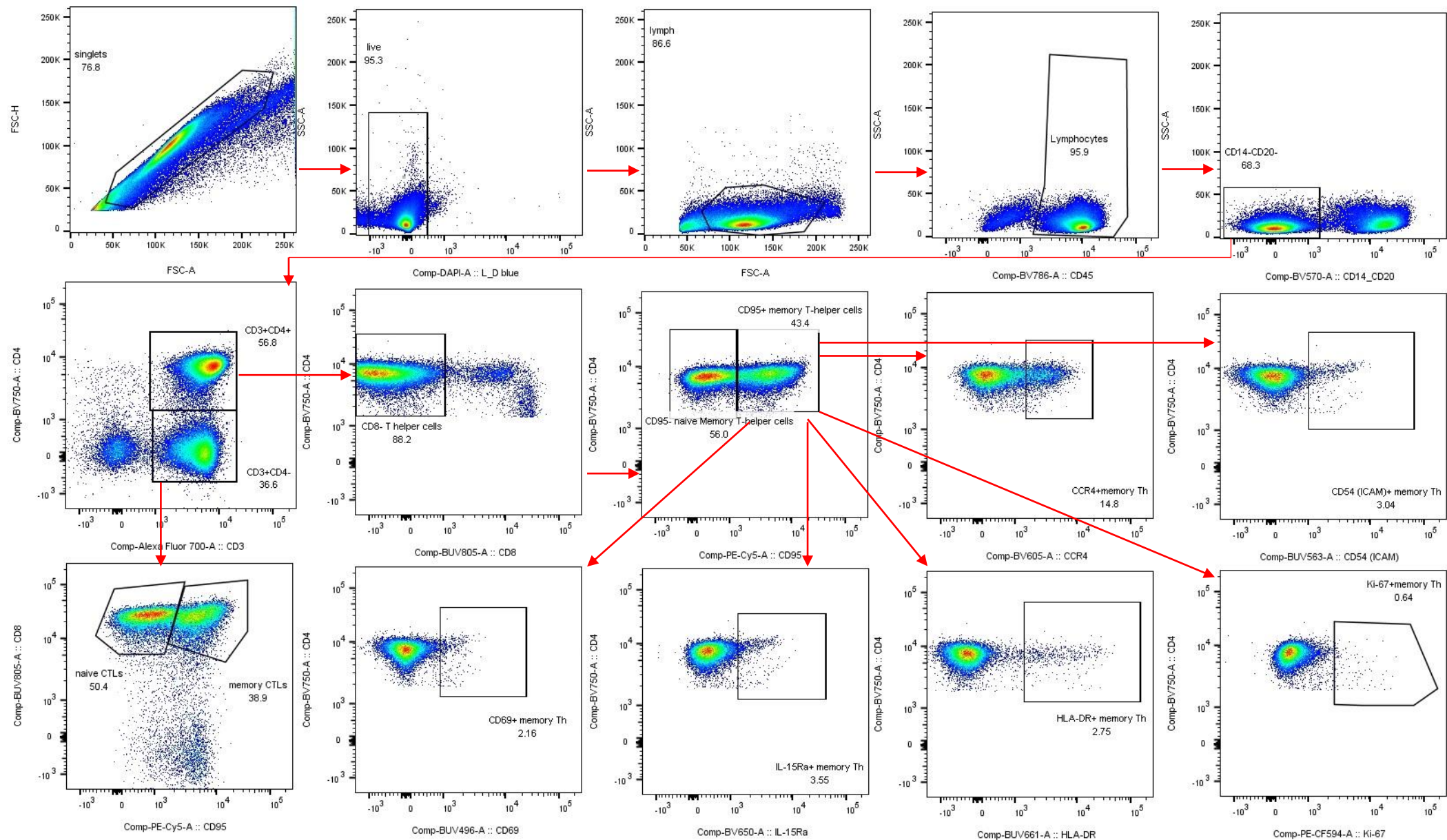

B

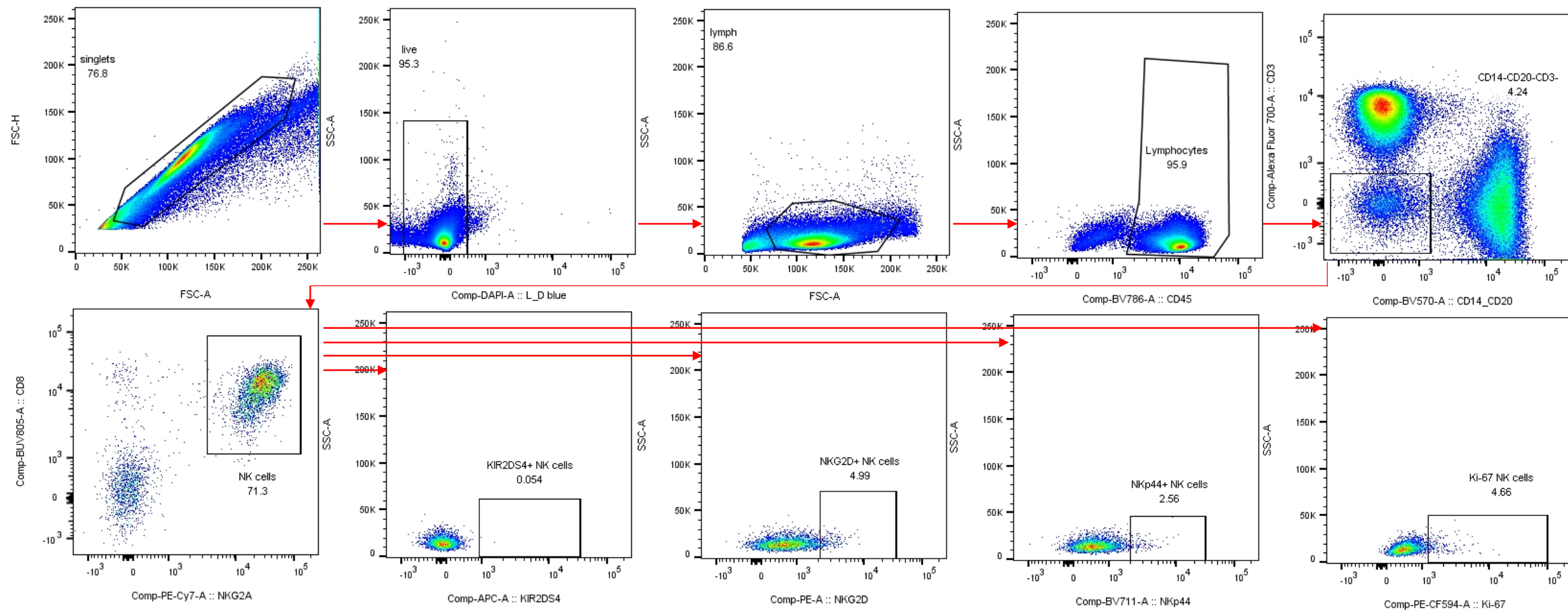

C

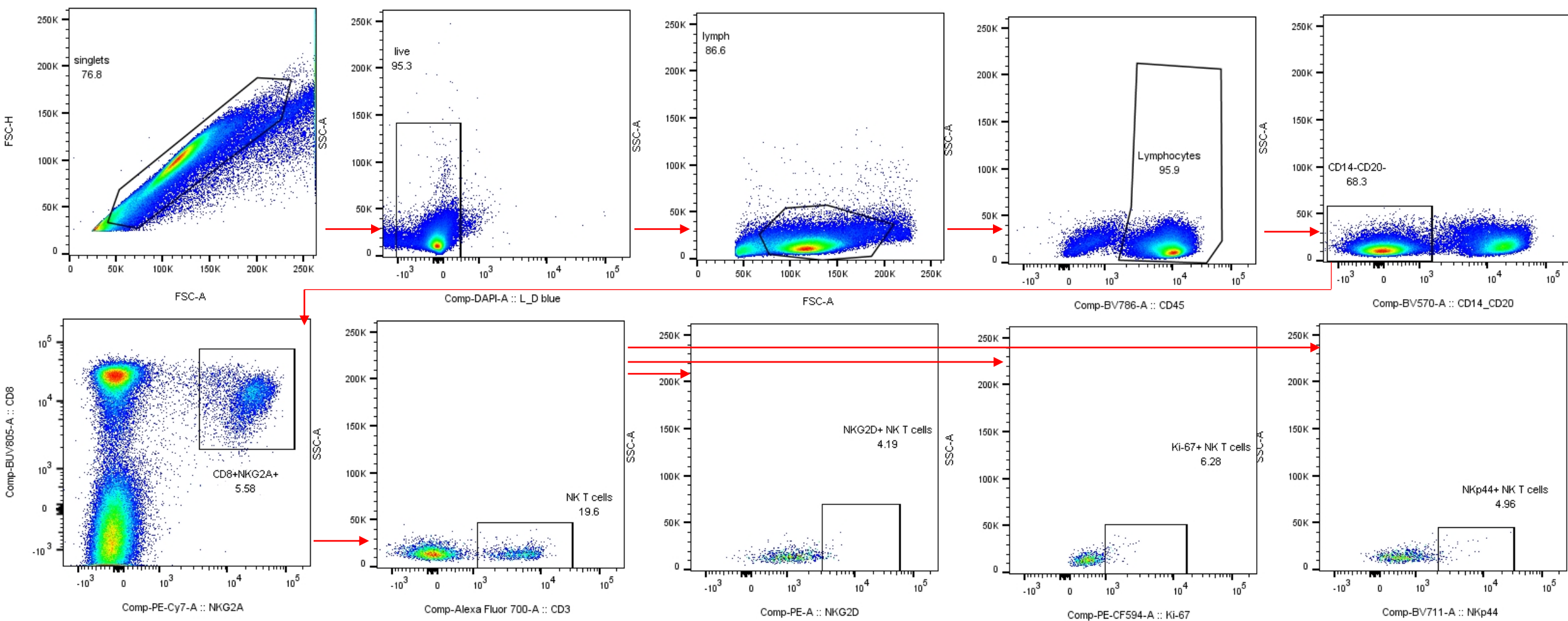

D

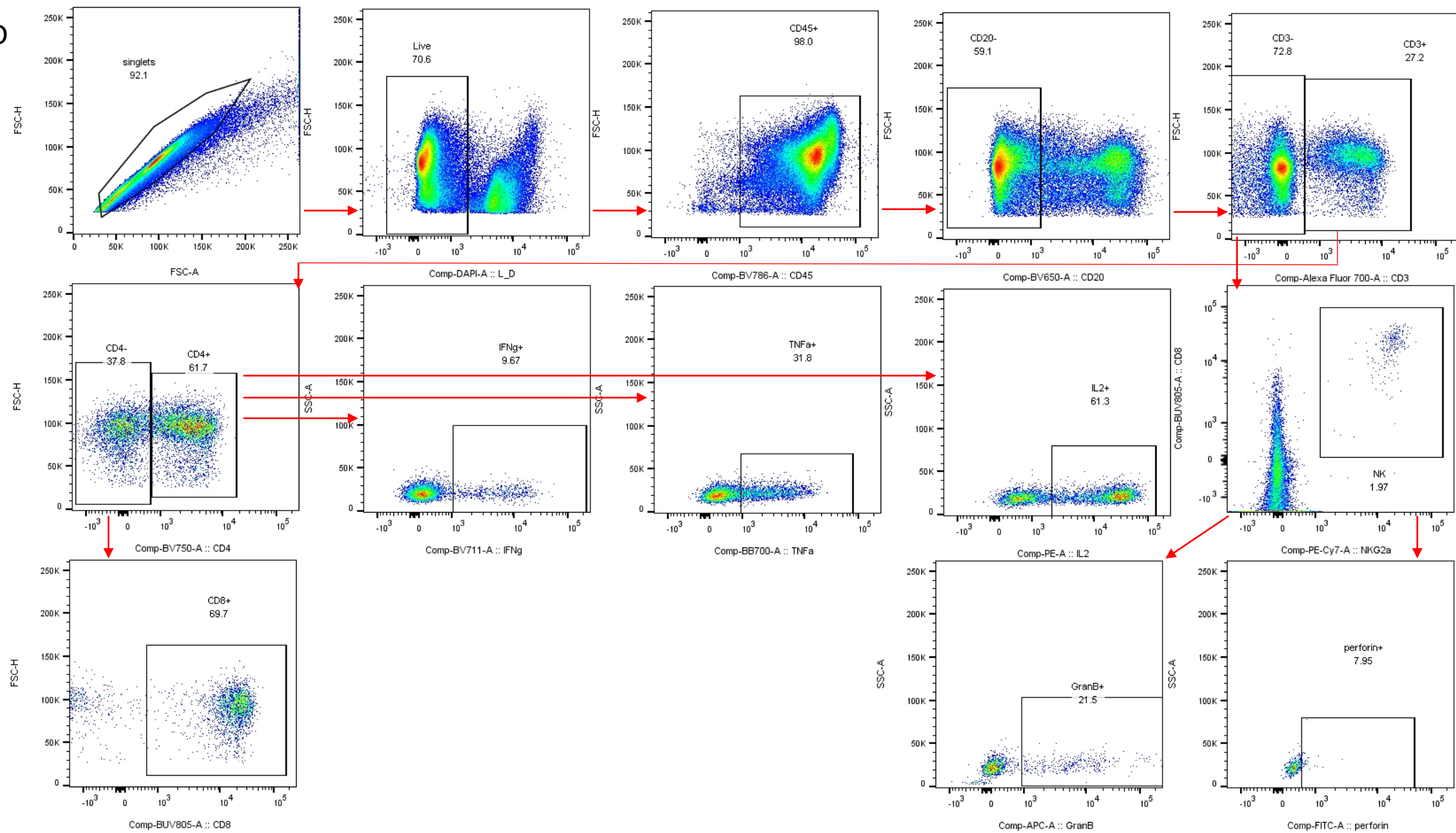

Supplement: Supplementary Figure 1 — Gating strategy. The gating strategy used for flow cytometry evaluation of the different (A) T cell subsets, (B) NK cells, (C) NKT cells, and (D) intracellular cytokine staining. [file Image_1.PDF]

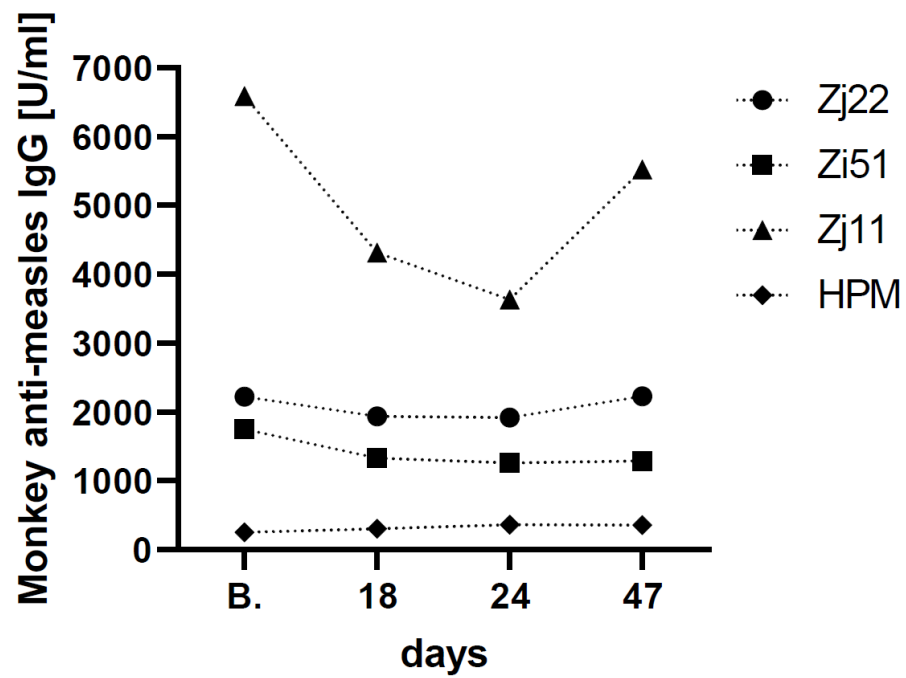

Supplement: Supplementary Figure 2 — Measles IgG antibody titers. Zi51 (square), Zj11 (triangle) and Zj22 (circle) were immunized for measles prior to the start of any study. HPM (diamond) was not immunized for measles. A graph of the monkey anti-measles IgG as measured by ELISA (Alpha Diagnostics International, Inc) is graphed for each animal at baseline (B.), day 18, day 24, and day 47 (23 days post Pom treatment). [file Image_2.PDF]
